# Salt Heat Treatment and Passivation to Improve the Corrosion Resistance of Nitinol (Ni-Ti)

**DOI:** 10.3390/ma14247789

**Published:** 2021-12-16

**Authors:** Inho Bae, Byung-Hoon Kim, Dong-Gon Kim, Ik-Bu Sohn, Seong-Won Yang

**Affiliations:** 1 Department of Dental Materials, School of Dentistry, Chosun University, 309 Pilmun-Daero, Dong-Gu, Gwangju 61452, Korea; inovation15@naver.com; 2 Department of Polymer Science and Engineering, Sunchon National University, Suncheon 57922, Korea; sibuki@hanmail.net; 3 Advanced Photonics Research Institute, Gwangju Institute of Science and Technology, 123 Cheomdangwagi-ro, Buk-Gu, Gwangju 61005, Korea; ibson@gist.ac.kr; 4 Department of Ophthalmology, College of Medicine, Chosun University Hospital, 365 Philmun-Daero, Dong-Gu, Gwangju 61453, Korea; smarteyes@hanmail.net

**Keywords:** nitinol, corrosion resistance, implantable materials, passivation, salt furnace

## Abstract

Corrosion of nitinol (NiTi) is a major factor in the failure of implantable materials. Recently, as the importance of corrosion of metals has increased, testing according to international guidelines is essential. The purpose of this study was to evaluate the corrosion resistance of NiTi wire through heat treatment and passivation process. In this study, NiTi wire used two commercially available products and a self-manufactured stent. Experimental consideration was carried out according to ASTM standards. Heat treatment was carried out in an air or a salt furnace, and the corrosion was measured after additional process, such as passivation and scratch tests. As a result, the metal potential was rapidly decreased in the air furnace group. On the other hand, the potential of wires was dramatically increased in the salt furnace group compared to the air furnace group. The dislocation decreased below the acceptance criteria (>600 mV) within 60 s of heat treatment time in the air furnace. Moreover, the potential was dramatically improved, even after only 20 min of passivation treatment (1076 mV, 442% compared to the non-passivated group), and it continued to rise until 180 min. This phenomenon was similarly observed in the group of self-manufactured stents. The potential slightly decreased by the scratch process (93.1%) was significantly reduced by the air furnace process (315 mV, 24.4% of the nontreated group). In the passivated group of the air furnace sample with reduced potential, the potential was restored to the level before the air furnace (scratch stage) (1032 mV). In conclusion, the heat treatment is preferably carried out in a salt furnace rather than an air furnace, and the passivation process can be an advantageous tool to improve corrosion resistance by suppressing the oxidation process.

## 1. Introduction

Nitinol (NiTi) is a metallic alloy of nickel (Ni) and titanium (Ti) in which the two elements are present in approximately equal atomic ratios. Due to the special properties of shape memory effect and superelasticity of Ni, it is widely used in various implantable materials, such as catheters, needles, staples, stents, dental wires, and orthopedic implants, and endodontic instruments [1,2,3,4,5,6]. However, NiTi alloys are more susceptible to corrosion due to ion release than stainless steel, cobalt–chromium or *β*-titanium. In particular, it has been found that the release of Ni ions decreases with time, but significant releases of Ni and Ti ions from dental alloys in corrosive environments are noted [7].

It was also reported that Ni release occurred after treatment with NiTi orthodontic archwires, bands, and brackets and was associated with an increase in Ni ion concentration in patients’ saliva, which persisted for 10 weeks, and then gradually decreased [8]. This phenomenon may cause various adverse reactions in the human body, such as foreign body reaction, allergy, and inflammation [9,10].

As an implantable material, nitinol must meet several requirements for biocompatibility combined with suitable mechanical properties and high corrosion resistance in chloride-rich media. The shape memory and superelastic properties of nitinol mentioned above, as well as the relatively low modulus of elasticity of this material (~80 GPa for austenitic form and ~30 GPa for martensitic form [11,12]), make it an excellent biomaterial in terms of mechanical properties. 

Generally speaking, nitinol is expensive, and difficult to process and machine compared to titanium, Co–Cr–Mo alloys, and even stainless steel [13,14]. However, it has been reported that procedures such as electropolishing, passivation in boiling water, and carbon layer deposition improved resistance to crevice corrosion [15], and, after chemical polishing, there is still a high Ni concentration on the surface of nitinol alloy [16]. On the other hand, it has been reported that electropolishing can form a Ni-deficient thin (about 10 nm) surface titanium oxide film and more effectively mitigates the outward diffusion of Ni ions, improving wettability, blood compatibility, and thrombus resistance [17]. 

Electropolishing also had better corrosion resistance than chemical etching, mechanical polishing, or thermal oxidation at 500 °C [18]. The oxide film formed by electropolishing contained less nickel than the other surface preparations, as evidenced by the lower nickel release [12]. It has been reported that the corrosion rate depends on the surface treatment and is the lowest for the passivation sample and the highest for the mechanical polishing sample [19]. Even after chemical polishing, a high Ni concentration was still present on the surface of the nitinol alloy, but in the case of electropolishing, a thin (about 10 nm) surface titanium oxide film free of Ni was formed. Electropolishing also more effectively mitigates the outward diffusion of Ni ions and improves wettability, blood compatibility, and thrombus resistance [20]. Furthermore, electropolishing showed better corrosion resistance than chemical etching, mechanical polishing, or thermal oxidation at 500 °C, and the oxide film formed by electropolishing showed less nickel release than other surface treatments [21]. The application of cyclic polarization techniques to a small size of material is covered by ASTM standard F2129, and the original 2001 version of the standard left users with a choice of solutions [22]. A simpler solution, 0.9% NaCl, is designated as the electrolyte in ASTM standard F746 for metal surgical implant materials [23], and has been occasionally used for periodic polarization tests as well. As many studies and the importance of corrosion resistance are increasing, international standards, such as ASTM, for human implantation of NiTi have been proposed, and further studies are essential. Additional processes, such as heat treatment to give NiTi advanced properties such as shape memory and superelasticity as a bioimplantable material, may reduce corrosion resistance. Therefore, the process that can recover the reduction in corrosion resistance that occurs during the post-processing process was to be presented in more detail and according to the international standard. The purpose of this study was to evaluate the corrosion resistance of NiTi used in nonvascular stents through a cyclic potential difference polarization test.

## 2. Materials and Methods

### 2.1. Materials and Preparation

Nitinol may experience surface corrosion and subsequent ion release due to electrochemical interactions occurring in the body. Thus, NiTi (50.8% at) wires purchased from Furukawa (Weilam Rhein, Germany) and NDC (Fremont, CA, USA) were used in this study. Before corrosion testing, both materials were pretreated to obtain surface properties similar to implantable materials. The nitinol wire was straightened and electropolished before the experiments. Test materials were sterilized and selected such that potential variations due to manufacturing were assessed. 

### 2.2. Experimental Consideration

The evaluation of the fracture pitting corrosion potential was measured. This evaluation was confirmed to be performed according to ASTM F2129: *Standard Test Method for Performing Cyclic Potential Difference Polarization Measurements to Determine Corrosion Sensitivity of Miniature Implant Devices* [24,25,26].

This evaluation was conducted taking the following into consideration:

- Factors such as shape or size that can affect surface finish, such as proper grinding of areas of high curvature, were considered.
- Test reports for official corrosion potential tests were conducted in accordance with ASTM F2129. For example, the test report included corrosion/static potential, breakdown potential, and polarization curves and reviewed any deviations from the ASTM F2129 standard (e.g., ASTM G5: Standard Reference Test for Potential Dynamic Bipolar Polarization Measurements) test setups that did not meet the criteria described in Methods.
- Results were evaluated against acceptance criteria, and acceptance criteria for formal corrosion testing applied legally marketed materials with a clinically good history of use (i.e., no history of corrosion-related fractures or adverse events related to nickel release).
- Alternatively, although data linking in vitro corrosion tests directly to in vivo corrosion results are lacking, conservative guidelines have been used to establish acceptance criteria.

### 2.3. Heat Treatment

The NiTis used in this study were cut to 7 mm in diameter and 100 mm in length using a laser cutting machine. Prior to the corrosion susceptibility testing, the NiTis were heated with air furnace at 540 for approximately 2 h to thermally grow the oxide layer to increase pitting corrosion susceptibility. 

### 2.4. Scratch Experiment

In order to simulate in vitro the friction between the struts of the wires occurring in the body, the friction between the NiTi wires was manually induced in phosphate buffered saline at 37 °C and pH 7.4 for more than 100 times. Each specimen was subjected to a passivation process and the degree of corrosion was examined. The surface morphology of the wire was visualized by optical microscope (Xi-CAM, Bestec Vision Co., Gunpo-si, Korea). 

### 2.5. Corrosion Testing

Cyclic potentiodynamic polarization corrosion testing was performed using an EG&G Princeton Applied Research potentiostat model 273A in accordance with ASTM F2129. The potentiostat was computer controlled with 352 SoftCorrIII-DC corrosion test software. A saturated calomel electrode (SCE) was used as the reference electrode for the potential, and two platinum auxiliary electrodes were used as counter electrodes. The solution was first degassed for 30 min during the test process before immersion of the test sample. Thereafter, the open circuit potential (OCP) was then monitored for 1 hr. The polarization of the test specimens was then initiated at 100 mV below the OCP at a voltage scan rate of 0.167 mV/sec. Testing was performed in Hank’s simulated physiological solution at an initial pH of 7.4 ± 0.1. All reactions were carried out at 37 ± 1 °C and the corrosion resistance of the material in terms of breakdown potential (Ebd) was characterized. 

### 2.6. Acceptance Criteria

Not only the ASTM F2129 test standard, but also the Food and Drug Administration (FDA) guidelines do not suggest acceptance criteria for corrosion resistance of implantable materials. In particular, the standards for corrosion testing of nonvascular stents are not clear. Therefore, the criterion was set according to the results of the corrosion resistance test from the related literature and the predicate material. In this study, it merely was established that the alloy was in an optimal state of corrosion resistance when the breakdown potential exceeded +600 mV (SCE) in PBS at 37 °C and, at breakdown potentials between +300 and +600 mV, the alloy is marginal, with a high confidence level.

## 3. Results

### 3.1. Cyclic Potentiodynamic Test

**Figure 1:**
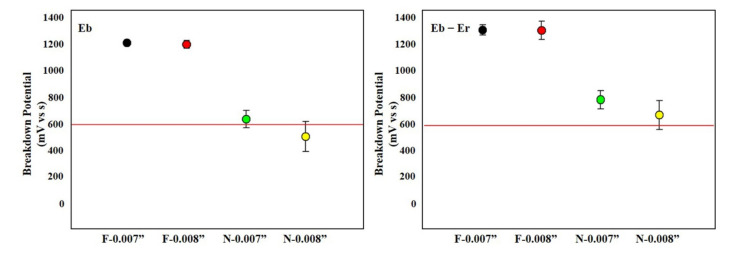
Cyclic potentiodynamic values of Furukawa and NDC wires without any treatment. Red lines indicate acceptance criteria in this study as mentioned in Materials and Methods section. ‘F’ and ‘N’ are acronyms for ‘Furukawa’ and ‘NDC’, respectively.

The corrosion test was performed according to the cyclic potentiodynamic test method of ASTM 2129. The electrochemical properties of NiTi materials were investigated in order to compare the current potential and constituent properties of the metals. Air furnace was performed at 500 °C with 0.007” and 0.008” of Furukawa and NDC NiTi each. As results show, Furukawa (no breakdown) of nitinol wire without heat treatment was superior to NDC (Eb > 500 mV). Although this phenomenon was similar for both 0.007” and 0.008” wire thickness, NDC (Eb-Er) satisfies the allowable standard (>600 mV) (Eb-Er: 1298 mV in 0.007” and 1292 mV in 0.008” of Furukawa wire vs. 802 mV in 0.007” and 662 mV in 0.008” of NDC wire (Figure 1)). 

### 3.2. Heat Treatment of Air Furnace

**Figure 2:**
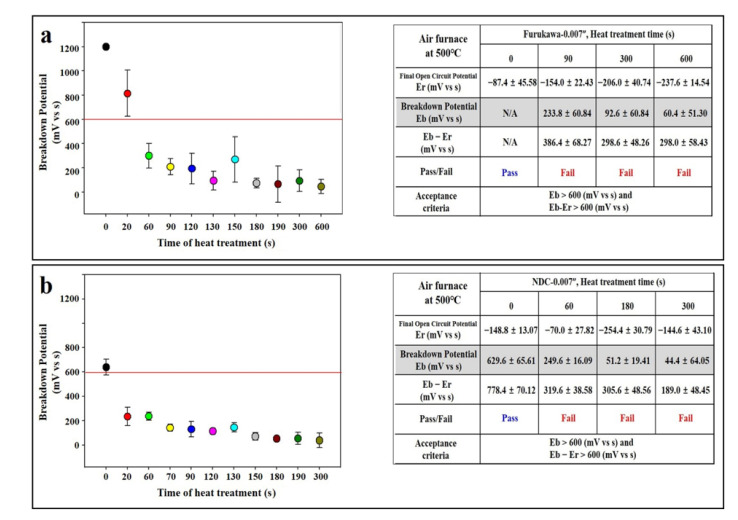
Heat treatment of wires in air furnace. (**a**) Furukawa and (**b**) NDC wire. Red lines indicate acceptance criteria in this study as mentioned in Materials and Methods section. ‘F’ and ‘N’ are acronyms for ‘Furukawa’ and ‘NDC’, respectively.

In order to give shape memory and superelasticity characteristics of NiTi, which are advantages as an implantable biomaterial, it is essential to undergo a heat treatment process. The test was performed with 0.007” in thickness wire. However, as shown in Figure 2, when the nature of NiTi was subjected to a heat treatment process, the metal potential rapidly decreased. This phenomenon occurred in both Furukawa (Figure 2a) and NDC (Figure 2b) wires. The dislocation decreased below the acceptance (600 mV) within 60 s of heat treatment time for Furukawa and 20 s for NDC (Furukawa; 1198 mV in 0 s, 812 mV in 20 s, and decreased less than 600 mV after 60 s vs. NDC; 619 mV in 0 s and decreased less than 600 mV after 20 s in NDC group).

### 3.3. Heat Treatment of Salt Bath

**Figure 3:**
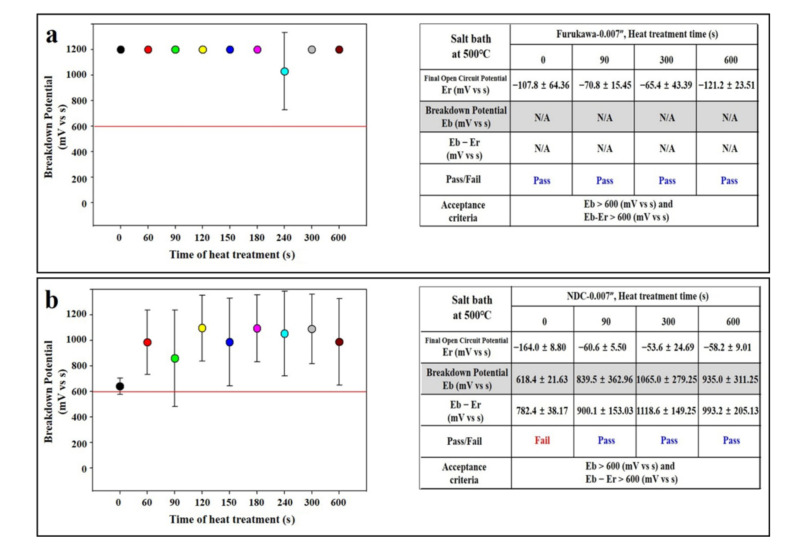
Heat treatment of wires in salt bath. (**a**) Furukawa and (**b**) NDC wire. Red lines indicate acceptance criteria in this study as mentioned in Materials and Methods section. ‘F’ and ‘N’ are acronyms for ‘Furukawa’ and ‘NDC’, respectively.

When heat treatment was carried out in an air furnace, both Furukawa and NDC showed a tendency to decrease to less than 600 mV. However, when heat treatment was performed in a salt furnace instead of an air furnace, although there were large deviations in the NDC group, the potential in both groups showed a tendency to improve (Furukawa; 1198 mV in 0 s, 1196 mV in 90 s, and there was maintained potential after 60 s of salt bath heat treatment (Figure 3a) vs. NDC: 623 mV in 0 s and increased over 600 mV after 60 s (Figure 3b)).

### 3.4. Passivation Experiment of Nitinol Wire

**Figure 4:**
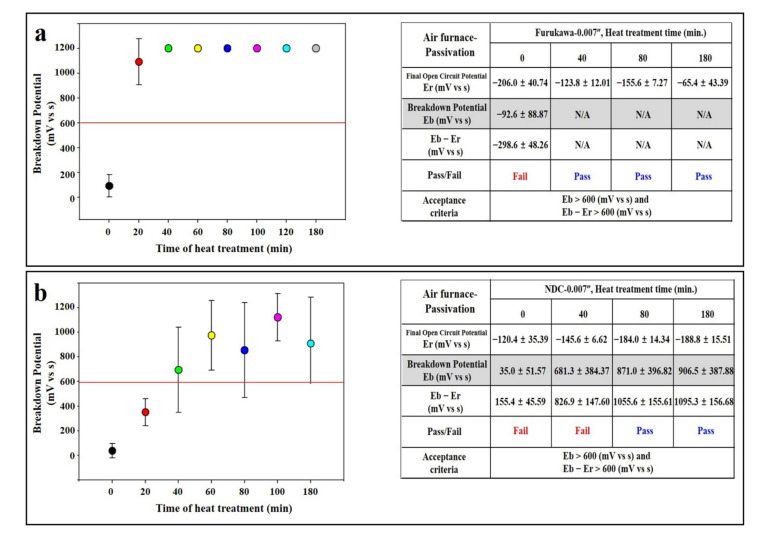
Passivation of wires after air furnace. (**a**) Furukawa and (**b**) NDC wire. Red lines indicate acceptance criteria in this study as mentioned in Materials and Methods section. ‘F’ and ‘N’ are acronyms for ‘Furukawa’ and ‘NDC’, respectively.

When heat treatment was performed in the air furnace, the potential of the Furukawa group was decreased below the acceptance (600 mV) at 60 s, and decreased to 60 mV (12% of the initial potential) at 600 s (Figure 2a). This phenomenon was similarly observed in the case of NDC as the potential decreased below the guideline (600 mV) at 20 s and decreased to 44 mV (15% of the initial potential) at 600 s (Figure 2b). Therefore, a passivation test was performed using a specimen that underwent an air furnace for 300 s. The experiment was conducted using a 0.007” in thickness specimen. As results show, the potential of Furukawa was dramatically improved, even after only 20 min of passivation treatment (1094 mV, 442% compared to the non-passivated group), and it continued to rise until 180 min (Figure 4a). Even in the case of the NDC sample, although it did not exceed the acceptance (600 mV), the potential was improved (368 mV), even after only 20 min passivation treatment. In addition, the potential was increased above the acceptance (673 mV) at 40 min passivation treatment and lasted until 180 min (Figure 4b).

### 3.5. Passivation Experiment of Stent

**Figure 5:**
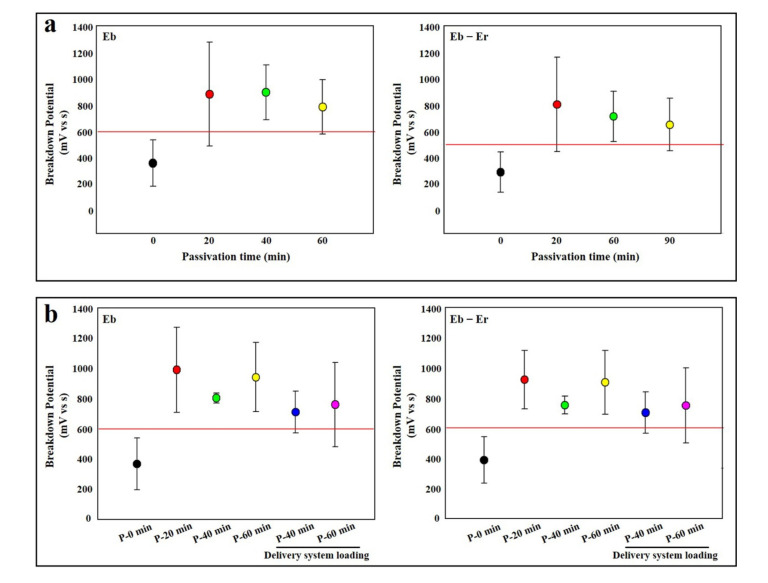
Passivation of stent after air furnace. (**a**) Fully covered stent and (**b**) double-covered stent. Red lines indicate acceptance criteria in this study as mentioned in Materials and Methods section.

In order to prove the effect of passivation in the prototype rather than the characteristics of the wire itself, covered stents were self-manufactured with bare NiTi, and the effect of passivation was confirmed using these. The samples were manufactured with fully or double-covered stents. As results show, the potential of the fully covered stent was recovered beyond the acceptance criteria (600 mV) at only 20 min of treatment (874 mV) and lasted until 90 s (Figure 5a). In addition, the potential of the double-covered stent was also restored to over 600 mV in 20 min and maintained over 600 mV, even through delivery material, three times (Figure 5b).

### 3.6. Scratch Test

**Figure 6:**
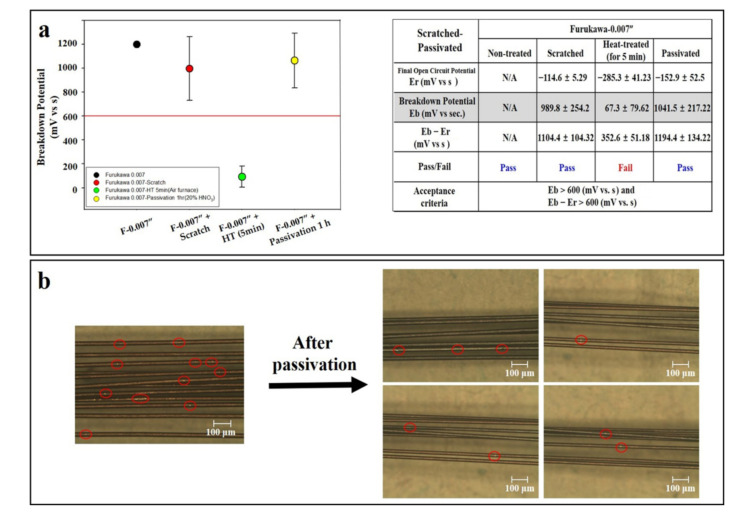
Corrosion test result after passivation treatment of scratched NiTi wire. (**a**) Potential change in wire by scratch, heat treatment in air furnace, passivation, and (**b**) optical microscopic images of wire surface morphology are represented. Red lines indicate acceptance criteria in this study as mentioned in Materials and Methods section. Red circles indicate areas of corrosion by friction. ‘F’ and ‘P’ are acronyms for ‘Furukawa’ and ‘passivation’, respectively.

In the case of biomaterials (especially stents) made of NiTi, friction between the wire struts inevitably occurs due to autonomous movement of blood vessels, muscles, organs, etc., or external physical force in the human body. This can be a major cause of corrosion and is an important consideration in the process of manufacturing biomaterials. Therefore, in this study, a scratch test was performed to implement it in vitro. As results show, the potential of the wire was slightly reduced due to the scratch process (Furukawa; 1208 mV to 989 mV in 0.007”). A follow-up study was carried out using a Furukawa 0.007” specimen to confirm a more drastic difference. The potential slightly decreased by the scratch process (93.1%) was significantly reduced by the air furnace process (67 mV, 6.7%). In the passivated group of the air furnace sample with reduced potential, the potential was restored to the level before the air furnace (1041 mV) (Figure 6a). This phenomenon was confirmed by microscopic observation (Figure 6b).

## 4. Discussion

Because metallic implants (especially NiTi) are prone to corrosion after implantation, corrosion resistance testing is an important evaluation to help ensure long-term implant durability in an in vivo environment [2,27]. There are several types of corrosion that can occur in implantable materials, such as galvanic, crevice, fretting, pitting, and metal ion release. This is recognized as an important factor in maintaining the functionality and safety of implantation materials in the human body. In this study, a method for improving corrosion resistance using a heat treatment method and passivation technology was proposed. 

The NiTi is a metal widely used as an implantable biomaterial due to its unique properties, such as shape memory and superelasticity. Despite these various advantages of NiTi, since it has been implanted in the human body for several decades, there remains a problem that corrosion can only occur due to chemical or physical reactions from the metal itself or external force [28,29,30]. For this reason, as the importance of corrosion of metals is emphasized, various guidelines mentioned above are presented, and research according to them is essential. Therefore, in this study, methods were proposed to recover the corrosion resistance that was reduced due to heat treatment to give nitinol’s shape memory and superelasticity function.

The NiTi wires used in this study were provided by Furukawa and NDC, and it was investigated that the potential of Furukawa was somewhat higher than that of NDC (Figure 1). Generally, NiTi is heat-treated in an air furnace for functionalization, which has a high probability of reacting with oxygen in the air. The titanium oxide film formed by the reaction of titanium contained in NiTi with oxygen in the air or dust during heat treatment significantly reduces the corrosion resistance of NiTi (Figure 2) [31].

In order to recover the corrosion resistance of NiTi, in this study, the heat treatment process for imparting functionality was performed in the salt furnace instead of the atmospheric furnace, and, as a result, the potential was continuously maintained above the guideline (Figure 3). It is considered that this is because the probability of titanium oxide film formation is reduced because direct contact with oxygen or dust in the air is blocked. Only the passivation process itself in the heat treatment process of the implantable material can suppress the formation of the titanium oxide film. This can be expected to have the effect of corrosion resistance due to the inhibition of titanium oxide formation, as mentioned above (Figure 4).

Although the above research was conducted with NiTi in the form of a wire, it is ultimately essential to implement it in the form of an implantable material. Therefore, in this study, a fully or double-covered stent was self-manufactured by using nitinol, and the passivation effect was demonstrated (Figure 5). In the case of final products as implantable materials, other metals, such as platinum, are comprised in NiTi, so it is judged that additional studies, such as galvanic or Ni release tests, are necessary. In addition to these chemical reactions, corrosion may also occur through physical reactions, such as scratching and friction. In particular, the stent continues to move in the body, which causes friction between the struts and may cause corrosion. To simulate this in vitro, an experiment was performed by artificially inducing a scratch on the wire surface. As results show, it was confirmed that corrosion was inhibited by passivation in the group induced by stretching (Figure 6). When the inside of the strut is exposed due to a scratch, ions are released, and it is directly corroded [32].

## 5. Conclusions

In conclusion, this study has shown that the advantage of heat treatment in a salt furnace and passivation on corrosion resistance of NiTi based on ASTM F2129. In this study, the potential of Furukawa and NDC’s NiTi wire of heat treatment in an air furnace was investigated. As a result, the potential of Furukawa was investigated to be somewhat higher than that of NDC. Both products have improved corrosion resistance through heat treatment in a salt furnace and passivation. These showed similar results in the scratched condition and prototype form. Taken together, it is preferable that the heat treatment process performed to impart functionality is carried out in a salt furnace rather than an air furnace, and it is suggested that corrosion resistance can be improved by going through a passivation subsequent process, even if it is unavoidably carried out in an air furnace. These were well implemented not only in the NiTi as a material, but also in the stent self-manufactured in the form of a prototype. Therefore, the above conclusions support the consideration of manufacturing commercial product that is economical and efficient and that can maintain functionality due to corrosion resistance.

## Data Availability

Data sharing not applicable.
